# Subtypes of Premorbid Metabolic Syndrome and Associated Clinical Outcomes in Older Adults

**DOI:** 10.3389/fmed.2021.698728

**Published:** 2022-02-11

**Authors:** Chu-Sheng Lin, Wei-Ju Lee, Shih-Yi Lin, Hui-Ping Lin, Ran-Chou Chen, Chi-Hung Lin, Liang-Kung Chen

**Affiliations:** ^1^Department of Family Medicine, Taichung Veterans General Hospital, Taichung, Taiwan; ^2^Center for Geriatrics and Gerontology, Taichung Veterans General Hospital, Taichung, Taiwan; ^3^Department of Post-Baccalaureate Medicine, College of Medicine, National Chung Hsing University, Taichung, Taiwan; ^4^Aging and Health Research Center, National Yang Ming Chiao Tung University, Hsinchu, Taiwan; ^5^Department of Geriatric Medicine, School of Medicine, National Yang Ming Chiao Tung University, Taipei, Taiwan; ^6^Department of Family Medicine, Taipei Veterans General Hospital Yuanshan Branch, Yi-Lan County, Taiwan; ^7^Department of Health, New Taipei City Government, New Taipei City, Taiwan; ^8^Chancellor's Office, National Yang Ming Chiao Tung University, Hsinchu, Taiwan; ^9^Center for Geriatrics and Gerontology, Taipei Veterans General Hospital, Taipei, Taiwan; ^10^Superintendent Office, Taipei Municipal Gan-Dau Hospital, Taipei, Taiwan

**Keywords:** metabolic syndrome, latent class analysis, older adults, cardiovascular risk, new onset diabetes mellitus

## Abstract

**Background:**

Metabolic syndrome has been shown to be a risk for new onset of cardiovascular disease (CVD) and type 2 diabetes. The subclasses of metabolic syndrome and any associated adverse health outcomes remain obscure. This study aimed to explore potential subtypes of metabolic syndrome, their associations with incidental diabetes, and any Major Adverse Cardiovascular Events (MACE).

**Methods:**

Data for the retrospective cohort study were extracted from the New Taipei City Elderly Health Examination Database in the years 2014 and 2016. Demographic data, status of metabolic syndrome, its components, and latent class analysis (LCA) were analyzed. All participants were aged 65 years and older, with those having a prior history of CVD, cerebrovascular disease, diabetes mellitus (DM), and currently taking medications for hypertension, diabetes, and dyslipidemia were excluded.

**Results:**

A total of 4,537 senior citizens were enrolled, with 2,207 (48.6%) of them identified as men. The prevalence of both metabolic syndrome and central obesity was increased with age. A 4-latent class model was fitted for participants diagnosed with metabolic syndrome. The central obesity (ABD)+ hyperglycemia (GLU)+ reduced HDL-C (HDL)+ high Blood Pressure (BP) group displayed the highest hazard ratio (HR) for predicting the new onset of diabetes, while the ABD+HDL+BP group showed a high risk for both CVD and MACE when compared after 2 years of follow-up.

**Conclusions:**

This epidemiological analysis demonstrated that the risks of developing new-onset diabetes, CVD, and MACE varied among the different subtypes of metabolic syndrome.

## Background

Older adults often suffer from cardiovascular diseases (CVDs), which are highly correlated with morbidity and mortality (1). Cardiovascular risks tend to cluster and act synergistically, thus resulting in a poor health prognosis with age (2). Metabolic syndrome is a risk factor for CVD morbidity and mortality, diabetes, and all-cause mortality (3). CVD is the main poor outcome resulting from metabolic syndrome, which has been shown in many studies (4–7). In one study, diabetes accounted for 10% of the population-attributable risk of a first myocardial infarction (8). Moreover, many studies have confirmed a strong relationship between metabolic syndrome and incident diabetes (4). Previous studies have clearly demonstrated consistent and strong associations between age and prevalence of metabolic syndrome (9–11).

In a meta-analysis that used the National Cholesterol Education Program (NCEP) or revised NCEP definitions, metabolic syndrome was associated with a two-fold increase in cardiovascular outcomes (7). In a study of middle-aged Japanese individuals followed up for a period of 14 years, the hazard ratio (HR) of CVD events was 1.86 (95% CI 1.32–2.62) in men with metabolic syndrome and 1.70 (95% CI 1.22–2.36) in women with metabolic syndrome (12).

Regardless of the definition used, metabolic syndrome was predictive of new-onset type 2 diabetes in many different populations (4). In a meta-analysis, metabolic syndrome, however defined, had a stronger association with incident diabetes than that previously demonstrated for coronary heart disease. The relative risk for incident diabetes was 3.5–5.2, while for CVD the relative risk was 1.5–2.0 (13).

With respect to metabolic syndrome components, in a population of individuals aged 43–84 years, the odds ratio of CVD was 1.95 in the group with one risk component, 2.05 in the group with two risk components, 2.70 in the group with three risk components, and 5.86 in the group with four or more risk components, based upon the WHO definition, but only the group with possessing more than 3 risk components showing a statistical significance (6). Several studies have reported the number of metabolic syndrome components associated with the risk of type 2 diabetes (4, 6, 14).

Impaired fasting glucose has been shown to be the strongest predictor in the development of type 2 diabetes among the components of metabolic syndrome (4, 15, 16). In a Framingham Offspring Study that involved participants with metabolic syndrome who had impaired fasting glucose, the relative risk of developing diabetes was 11, whereas among those with metabolic syndrome who did not have impaired fasting glucose, the relative risk of developing diabetes was 5 (14).

The effects of metabolic syndrome components may vary among its subclasses. Latent class analysis (LCA) may be capable of identifying distinct profiles in individuals based on their presentation of metabolic syndrome components (17). The purpose of LCA is to classify similar individuals into groups. A latent cluster consists of homogeneous individuals in terms of the observed variables, where different latent clusters represent the unobserved heterogeneity among individuals with respect to these observed variables (17, 18). The WHO describes metabolic syndrome as a pre-morbid condition, rather than a clinical diagnosis for primary care and preventive services (19), and suggests excluding individuals with diabetes mellitus (DM) or CVD from those included in the definition of metabolic syndrome. Therefore, the main purpose of this study was to evaluate the subclasses of metabolic syndrome and its poor health outcomes that included the new onset of diabetes, stroke, and CVD among Taiwanese aged over 65 years who had no prior history of CVD, cerebral vascular disease, hypertension, diabetes, or hyperlipidemia.

## Methods

### Subjects

Data for the retrospective cohort study were extracted from the New Taipei City Elderly Health Examination Database (NTCHD) in the years 2014 and 2016 and were obtained for analysis with authorization from the New Taipei City Government. The NTCHD aims to provide annual health examinations for early detection of health conditions and to promote senior health. All residences living in New Taipei City aged ≥65 years were encouraged to voluntarily receive this free service. All participants received a face-to-face interview with physicians in order to collect demographic characteristics, past medical and personal history, lifestyle factors, and functional data. All biomarkers associated with metabolic syndrome were collected from overnight fasting participants. Details of recruitment and data collection procedures have been described elsewhere (20). For the year 2014, all participants with a medical history of CVD, cerebrovascular disease, DM, hypertension, hyperlipidemia, or the use of medications treating those diseases were excluded from the analysis.

**Figure 1:**
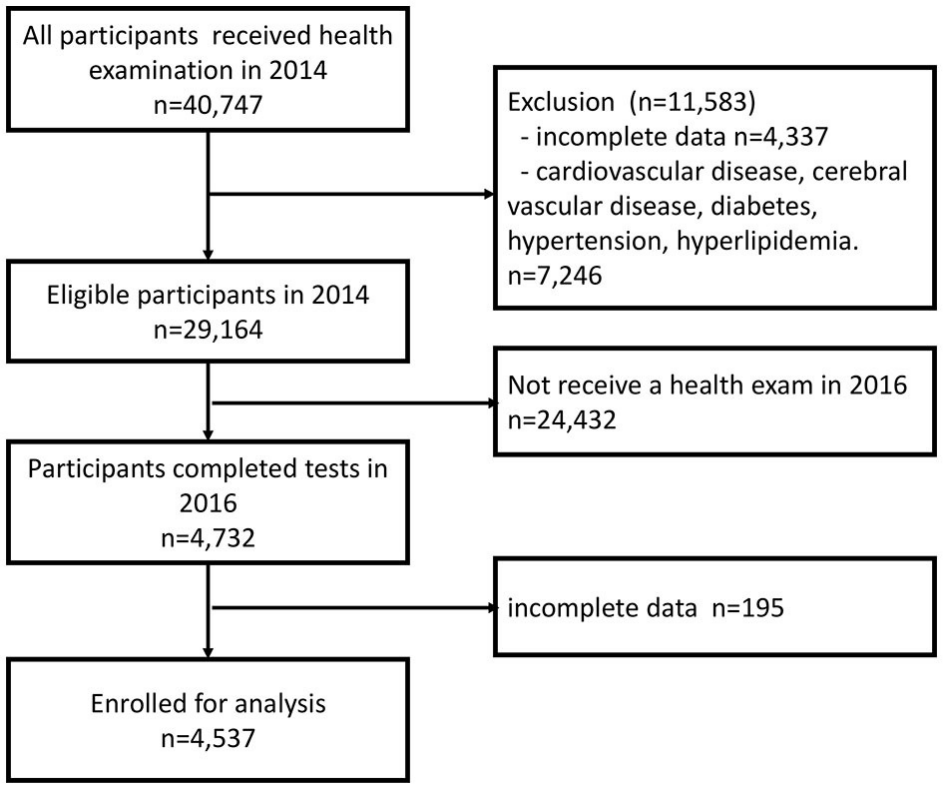
Flow-chart of inclusion and exclusion criteria.

In 2014, 40,747 individuals aged 65 years or older were participating in the New Taipei City Elderly Health Examination Program. We excluded 4,337 participants who provided incomplete data for MS (Supplemental Table S1), while also excluding 7,246 participants who had a prior medical history of CVD, cerebrovascular disease, DM, hypertension, hyperlipidemia, or use of medications for those diseases. Twenty-four thousand four hundred thirty-two participants either did not attend health examinations in the year 2016 or their screening time was uncertain. Additionally, in the year 2016, 195 participants provided incomplete data of MS. Eventually, 4,537 participants were included for analysis (Figure 1).

All participants signed a written consent to authorize the use of their data for research and policy purposes. The New Taipei City Government removed all potentially identified data. The New Taipei City Government also approved the use of the anonymized database and waived the requirement for Institutional Review Board approval.

### Definition of Outcomes

Data surrounding the new onset of DM, cerebrovascular disease, and CVD were then obtained from medical records in 2016. All data were taken from self-reported physician diagnoses.

### Definition of Metabolic Syndrome

In this study, the modified Third Report of the National Cholesterol Education Program's Adult Treatment Panel was used to define metabolic syndrome. Subjects who met three or more of the following five criteria were defined as having metabolic syndrome (21, 22):

(1) high blood pressure (BP): BP of at least 130/85 mm Hg,
(2) hypertriglyceridemia (TRI): serum triglyceride levels of at least 150 mg/dl,
(3) reduced high-density lipoprotein cholesterol (HDL-C) (HDL): HDL-C levels <40 mg/dl in men and <50 mg/dl in women,
(4) hyperglycemia (GLU): a fasting plasma glucose level of 100 mg/dl or more,
(5) central obesity (ABD): waist circumference ≥90 cm in men or ≥80 cm in women.

### Other Variables (Lifestyle Factors)

Smoking was defined as having used tobacco during the previous 6 months. Drinking was defined as having consumed alcohol during the previous 6 months. Exercise habit was defined as having exercised ≥150 min per week during the previous 2 weeks. The above information was taken from the questionnaire section of the New Taipei City Elderly Health Examination.

### Statistical Analysis

Latent class analysis was used to identify distinct subgroups of participants with different metabolic syndrome phenotypes. LCA is a subset of structural equation modeling, which is used to detect homogeneous subgroups within a larger heterogeneous population (23–25). The subtypes, termed “latent classes”, may represent disease entities or patterns of association in the respective phenotypes. LCA was therefore used to determine the number of classes or subgroups (26). We then constructed LCA models with the number of subclasses varying from two to four. These models were examined for fit, using the Akaike Information Criterion (AIC) (27) and the Bayesian Information Criterion (BIC) (28). Lower values of AIC and BIC indicate a better-fitted model (29, 30).

In the present study, statistical analysis was performed using commercial statistical software, SPSS 22.0 (IBM Corp, Chicago, IL, USA) and R version 2.15.3 (R Foundation for Statistical Computing, Vienna, Austria). Continuous variables in the text and tables are expressed as mean ± SD, and categorical data are expressed as numbers (percentage). Comparisons between continuous variables were performed by a paired *t*-test and independent *t*-test when appropriate, while comparisons between categorical data were carried out by the McNemar test, χ^2^ test, or Fisher's exact test when appropriate. The cumulative incidence rate was used for new onset diseases. Cox proportional hazards regression models were used to estimate HRs. For all tests, a *p* < 0.05 (two-tailed) was considered statistically significant.

## Results

When comparing the included and excluded participants in 2014, there is only a difference in age between the two groups, with no difference in gender, health behavior, or weight (Supplemental Table S1). A total of 4,537 senior citizens aged 65 years or older who participated in the New Taipei City Elderly Health Examination Program in both 2014 and 2016 were enrolled. At the end of the 2-year follow-up period, the body mass index (BMI) of the participants (24.49 ± 3.47 vs. 24.41 ± 3.42, *p* = 0.004), weight (60.99 ± 10.38 vs. 60.78 ± 10.47, *p* < 0.001), diastolic BP (DBP) (78.14 ± 10.91 vs. 77.21 ± 11.26, *p* < 0.001), and triglycerides (117.03 ± 66.71 vs. 114.85 ± 67.83, *p* = 0.024) had all decreased. Alternatively, waist circumference (83.20 ± 9.72 vs. 84.59 ± 9.66, *p* < 0.001), systolic BP (SBP) (133.91 ± 18.19 vs. 134.63 ± 18.52, *p* = 0.007), pulse pressure (55.77 ± 13.82 vs. 57.42 ± 13.74, *p* < 0.001), fasting plasma glucose (104.02 ± 21.92 vs. 104.89 ± 25.17, *p* = 0.009), central obesity (43.2% vs. 48.2%, *p* < 0.001), and prevalence of metabolic syndrome (32.8% vs. 34.6%, *p* = 0.012) had all increased over the 2-year period (Table 1).

**Table 1:** Demographic data of study participants in 2014 and 2016.

	**2014 (*****n*** **=** **4,537)**		**2016 (*****n*** **=** **4,537)**		***p*-value**
	***n***	**%**	***n***	**%**	
**Age**	71.75	±5.93	73.75	±5.93	–
**Gender**					1.000
Male	2,207	(48.6%)	2,207	(48.6%)	
Female	2,330	(51.4%)	2,330	(51.4%)	
**Smoking**	309	(6.8%)	297	(6.5%)	0.119
**Drinking**	547	(12.1%)	581	(12.8%)	0.862
**Exercise**	3,237	(71.3%)	3,462	(76.3%)	0.930
**BMI**	24.49	±3.47	24.41	±3.42	0.004^**^
**Height (cm)**	157.67	±8.15	157.59	±8.12	0.063
**Weight (kg)**	60.99	±10.38	60.78	±10.47	<0.001^**^
**Waist (cm)**	83.20	±9.72	84.59	±9.66	<0.001^**^
**SBP (mmHg)**	133.91	±18.19	134.63	±18.52	0.007^**^
**DBP (mmHg)**	78.14	±10.91	77.21	±11.26	<0.001^**^
**Pulse pressure (mmHg)**	55.77	±13.82	57.42	±13.74	<0.001^**^
**Fasting plasma glucose (mg/dl)**	104.02	±21.92	104.89	±25.17	0.009^**^
**Triglycerides (mg/dl)**	117.03	±66.71	114.85	±67.83	0.024^*^
**HDL cholesterol (mg/dl)**	55.61	±15.66	55.60	±15.78	0.922
**Waist (cm) male>90, female>80**	1,959	(43.2%)	2,188	(48.2%)	<0.001^**^
**High blood pressure:** **≧130/85 mm Hg**	2,768	(61.0%)	2,826	(62.3%)	0.119
**High fasting blood sugar:** **≧100 mg/dl**	2,196	(48.4%)	2,199	(48.5%)	0.953
**Hypertriglyceridemia:** **≧150 mg/dl**	969	(21.4%)	946	(20.9%)	0.453
**Low HDL cholesterol:** ** <40 mg/dl in men and:** ** <50 mg/dl in women**	1,103	(24.3%)	1,090	(24.0%)	0.660
**Metabolic syndrome**					0.012^*^
No	3,051	(67.2%)	2,968	(65.4%)	
Yes	1,486	(32.8%)	1,569	(34.6%)	
**Number of metabolic syndrome components**					0.158
0	570	(12.56%)	526	(11.59%)	
1	1,126	(24.81%)	1,108	(24.42%)	
2	1,355	(29.86%)	1,334	(29.40%)	
3	911	(20.08%)	961	(21.18%)	
4	449	(9.9%)	450	(9.92%)	
5	126	(2.78%)	158	(3.48%)	

*McNemar test*.

* *p < 0.05*,

** *p < 0.01. The bold values represent statistically significant*.

In 2014, 1,486 participants were diagnosed with metabolic syndrome, i.e., 679 (45.7%) who were male. The most common metabolic syndrome component combinations were GLU+BP+ABD (25.8%), followed by GLU+HDL-C+BP+ABD (10%), and GLU+TRI+HDL-C+BP+ABD (8.5%). Among GLU+TRI+HDL-C+BP+ABD (8.5%), GLU+TRI+HDL-C+ABD (3.9%), GLU+TRI+BP (4.9%), and GLU+BP+ABD (25.8%), a gender difference was noted (Table 2).

**Table 2:** Prevalence of metabolic syndrome component combinations in participants with metabolic syndrome in 2014.

	**Total (*n = * 1,486)**		**Male (*n =* 679)**		**Female (*n =* 807)**		***p*-value**
	***n***	**%**	***n***	**%**	***n***	**%**	
GLU+ TRI+HDL-C+BP+ABD	126	(8.5%)	46	(6.8%)	80	(9.9%)	0.031^*^
GLU+ TRI+HDL-C+BP	68	(4.6%)	32	(4.7%)	36	(4.5%)	0.817
GLU+ TRI+HDL-C+ABD	58	(3.9%)	19	(2.8%)	39	(4.8%)	0.044^*^
GLU+HDL-C+BP+ABD	149	(10.0%)	59	(8.7%)	90	(11.2%)	0.115
GLU+ TRI+BP+ABD	107	(7.2%)	58	(8.5%)	49	(6.1%)	0.067
TRI+HDL-C+BP+ABD	67	(4.5%)	23	(3.4%)	44	(5.5%)	0.056
GLU+ TRI+HDL-C	40	(2.7%)	21	(3.1%)	19	(2.4%)	0.381
GLU+ TRI+BP	73	(4.9%)	48	(7.1%)	25	(3.1%)	<0.001^**^
GLU+ TRI+ABD	42	(2.8%)	17	(2.5%)	25	(3.1%)	0.491
GLU+HDL-C+BP	82	(5.5%)	45	(6.6%)	37	(4.6%)	0.086
GLU+HDL-C+ABD	66	(4.4%)	29	(4.3%)	37	(4.6%)	0.770
GLU+BP+ABD	384	(25.8%)	196	(28.9%)	188	(23.3%)	0.015^*^
TRI+HDL-C+BP	42	(2.8%)	22	(3.2%)	20	(2.5%)	0.377
TRI+HDL-C+ABD	30	(2.0%)	13	(1.9%)	17	(2.1%)	0.793
TRI+BP+ABD	73	(4.9%)	26	(3.8%)	47	(5.8%)	0.076
HDL-C+BP+ABD	79	(5.3%)	25	(3.7%)	54	(6.7%)	0.004^**^

*Chi-square test*.

* *p < 0.05*,

** *p < 0.01*.

*ABD, central obesity; GLU, hyperglycemia; HDL, reduced HDL-C; Bp, high blood pressure; TRI, hypertriglyceridemia. The bold values represent statistically significant*.

The four-class model had the lowest AIC and BIC and should be the most suitable model in the LCA (Table 3).

**Table 3:** Latent class model fit statistics of participants with metabolic syndrome in 2014.

	**2-class model**	**3-class model**	**4-class model**
AIC	8,029	7,820	7,759
BIC	8,087	7,910	7,875

In the four-class model, ABD+HDL+BP, ABD+GLU+HDL+BP, ABD+GLU+HDL+BP+ TRI, and ABD+GLU+BP were identified, with the prevalence of those four classes being 5.3, 20.0, 48.8, and 25.8%, respectively (Table 4). To compare the demographic differences between the four-class model and other metabolic syndromes, the participants with metabolic syndrome were then grouped into 5 classes: ABD+HDL+BP, ABD+GLU+HDL+BP, ABD+GLU+HDL+BP+TRI, ABD+GLU+BP, and others. The ABD+GLU+BP class was the oldest group, with predominantly male participants. Health behaviors of the participants showed no statistically significant differences among the classes (Table 5).

**Table 4:** Conditional probabilities of meeting criteria within latent metabolic syndrome 4-class model of participants with metabolic syndrome in 2014.

**Rho estimate**	**4-class model**			
	**Class 1**	**Class 2**	**Class 3**	**Class 4**
Class prevalence (%)	5.3	20.0	48.8	25.8
ABD	1.000	0.724	0.693	1.000
GLU	0.000	1.000	0.708	1.000
HDL	1.000	1.000	0.593	0.000
BP	1.000	0.778	0.766	1.000
TRI	0.005	0.000	1.000	0.000

**Table 5:** Comparison of demographic data in study participants by subtypes of four-class model and others metabolic syndrome.

	**Non-MS**		**MS**		**ABD HDL BP**		**ABD GLU HDL BP**		**ABD GLU HDL BP TRI**		**ABD GLU BP**		**Others MS**		***p*-value**
	**(*n =* 3,051)**		**(*n =* 1,486)**		**(*n =* 79)**		**(*n =* 149)**		**(*n =* 126)**		**(*n =* 384)**		**(*n =* 748)**		
	***n*** **%**		***n*** **%**		***n*** **%**		***n*** **%**		***n*** **%**		***n*** **%**		***n*** **%**		
**Age**	71.66 ± 5.85		71.94 ± 6.09		72.92 ± 6.21		72.16 ± 6.31		71.54 ± 6.14		73.00 ± 6.40		71.32 ± 5.77		<0.001**
**Gender**															<0.001**
Male	1,528 (50.1%)		679 (45.7%)		25 (31.6%)		59 (39.6%)		46 (36.5%)		196 (51.0%)		353 (47.2%)		
Female	1,523 (49.9%)		807 (54.3%)		54 (68.4%)		90 (60.4%)		80 (63.5%)		188 (49.0%)		395 (52.8%)		
**Smoking**	204 (6.9%)		105 (7.4%)		4 (5.2%)		9 (6.2%)		10 (8.3%)		23 (6.2%)		59 (8.3%)		0.684
**Drinking**	361 (12.3%)		186 (13.1%)		6 (7.8%)		14 (9.7%)		14 (11.7%)		57 (15.4%)		95 (13.5%)		0.283
**Exercise**	2,203 (77.3%)		1,034 (73.9%)		56 (72.7%)		105 (72.9%)		84 (72.4%)		272 (74.1%)		517 (74.3%)		0.264
**Waist (cm) male>90 female>80**	778 (25.5%)		1,181 (79.5%)		79 (100.0%)		149 (100.0%)		126 (100.0%)		384 (100.0%)		443 (59.2%)		<0.001**
**High blood pressure:** **≧130/85 mm Hg**	1,518 (49.8%)		1,250 (84.1%)		79 (100.0%)		149 (100.0%)		126 (100.0%)		384 (100.0%)		512 (68.4%)		<0.001**
**High fasting blood sugar:** **≧100 mg/dl**	1,001 (32.8%)		1,195 (80.4%)		0 (0.0%)		149 (100.0%)		126 (100.0%)		384 (100.0%)		536 (71.7%)		<0.001**
**Hypertriglyceridemia:** **≧150 mg/dl**	243 (8.0%)		726 (48.9%)		0 (0.0%)		0 (0.0%)		126 (100.0%)		0 (0.0%)		600 (80.2%)		<0.001**
**Low HDL cholesterol:** ** <40 mg/dl in men and:** ** <50 mg/dl in women**	296 (9.7%)		807 (54.3%)		79 (100.0%)		149 (100.0%)		126 (100.0%)		0 (0.0%)		453 (60.6%)		<0.001**

Chi-square test. *p <0.05, **p <0.01. The bold values represent statistically significant.

After 2 years of follow-up, cumulative incidence rates in new-onset DM, stroke, CVD, and Major Adverse Cardiovascular Events (MACE) were 7.51, 0.89, 6.9, and 7.71%, respectively. The participants without any metabolic syndrome components were defined as the reference group. The new onset of diabetes appeared to be correlated with the number of metabolic syndrome components. There was a significantly greater prevalence of new-onset diabetes among participants with 2 metabolic syndrome components and a diagnosis of metabolic syndrome. Among the subclasses of metabolic syndrome, ABD+GLU+HDL+BP had an HR of 9.07 (95% CI: 5.12–16.06) for predicting the new onset of diabetes, which was higher than that of the other classes. Regarding stroke, CVD, and MACE, there were no statistically significant differences among these subclasses, except for ABD+HDL+BP, which had a high risk of CVD and MACE, with an HR of 1.95 (95% CI: 1.08–3.51) and 1.94 (95% CI: 1.10–3.42), respectively, in crude analysis. After adjusting for age, gender, and family history of CVD, there was no change in risk for CVD and MACE in the ABD+HDL+BP group (Table 6).

**Table 6:** Poor health outcomes among different metabolic syndrome states.

**Metabolic syndrome state**	**Poor health outcome**											
	**DM**			**Stroke**			**CVD**			**MACE**		
	**Number/event**	**HR 95%CI**	***p***	**Number/event**	**HR 95%CI**	***p***	**Number/event**	**HR 95%CI**	***p***	**Number/event**	**HR 95%CI**	***p***
MS component 0	570/16	Ref.		570/6	Ref.		570/62	Ref.		570/67	Ref.	
MS component 1	1126/53	1.65 (0.94–2.89)	0.078	1126/13	1.04 (0.39–2.73)	0.944	1,126/118	0.93 (0.68–1.27)	0.643	1,126/130	0.95 (0.70–1.27)	0.717
MS component 2	1355/156	3.88 (2.32–6.50)	<0.001**	1355/21	1.27 (0.51–3.17)	0.611	1,355/155	0.94 (0.70–1.26)	0.669	1,355/167	0.94 (0.70–1.25)	0.649
MS	1486/307	7.19 (4.35–11.89)	<0.001**	1486/24	1.55 (0.63–3.80)	0.338	1,486/191	1.06 (0.80–1.42)	0.684	1,486/211	1.1 (0.83–1.45)	0.507
ABD HDL BP	79/4	2.4 (0.79–7.29)	0.124	79/1	1.43 (0.17–12.33)	0.746	79/14	1.93 (1.05–3.55)	0.033*	79/15	1.89 (1.05–3.40)	0.032*
ABD GLU HDL BP	149/46	9.5 (5.34–16.90)	<0.001**	149/2	1.18 (0.23–5.95)	0.84	149/21	1.01 (0.61–1.68)	0.964	149/23	1.02 (0.63–1.66)	0.923
ABD GLU HDL BP TRI	126/34	8.41 (4.62–15.31)	<0.001**	126/1	0.68 (0.08–5.64)	0.718	126/22	1.29 (0.79–2.12)	0.312	126/22	1.19 (0.73–1.95)	0.48
ABD GLU BP	384/80	6.69 (3.89–11.52)	<0.001**	384/6	1.35 (0.42–4.31)	0.615	384/66	1.26 (0.88–1.81)	0.198	384/69	1.23 (0.87–1.74)	0.239
Others MS	748/143	6.9 (4.12–11.58)	<0.001**	748/14	1.82 (0.70–4.74)	0.22	748/68	0.84 (0.59–1.19)	0.323	748/82	0.94 (0.68–1.30)	0.713
MS component 0	570/16	ref.		570/6	Ref.		570/62	Ref.		570/67	Ref.	
MS component 1	1,126/53	1.48 (0.84–2.60)	0.174	1126/13	1.3 (0.46–3.66)	0.616	1126/118	0.97 (0.70–1.33)	0.835	1,126/130	0.95 (0.70–1.29)	0.926
MS component 2	1,355/156	3.46 (2.07–5.80)	<0.001**	1355/21	1.52 (0.57–4.08)	0.404	1355/155	0.96 (0.70–1.30)	0.773	1,355/167	0.9 (0.66–1.22)	0.732
MS	1,486/307	6.32 (3.82–10.47)	<0.001**	1486/24	1.56 (0.59–4.13)	0.374	1486/191	1.09 (0.81–1.46)	0.585	1,486/211	1.12 (0.84–1.48)	0.453
ABD HDL BP	79/4	2.14 (0.71–6.42)	0.174	79/1	1.78 (0.21–15.22)	0.6	79/14	2.07 (1.15–3.75)	0.016*	79/15	2.05 (1.16–3.63)	0.014*
ABD GLU HDL BP	149/46	7.59 (4.25–13.58)	<0.001**	149/2	1.31 (0.25–6.73)	0.75	149/21	0.91 (0.54–1.54)	0.723	149/23	0.94 (0.57–1.56)	0.825
ABD GLU HDL BP TRI	126/34	7.1 (3.80–13.29)	<0.001**	126/1	–		126/22	1.24 (0.72–2.13)	0.432	126/22	1.15 (0.68–1.96)	0.601
ABD GLU BP	384/80	6.09 (3.55–10.44)	<0.001**	384/6	1.3 (0.37–4.50)	0.681	384/66	1.25 (0.87–1.81)	0.224	384/69	1.22 (0.86–1.74)	0.261
Others MS	748/143	6.27 (3.73–10.55)	<0.001**	748/14	1.92 (0.68–5.38)	0.274	748/68	0.96 (0.67–1.38)	0.833	748/82	1.04 (0.74–1.47)	0.802

*Adjust for gender and age*.

Adjusted for family history of cardiovascular disease. The bold values represent statistically significant. *p <0.05, **p <0.01.

## Discussion

The results of this study show that the prevalence of metabolic syndrome and central obesity increases with age. A 4-latent class model was used to classify participants with metabolic syndrome. The ABD+GLU+BP class was the largest and oldest group among the 4 LCA models. ABD+GLU+HDL+BP had the highest HR for predicting the new onset of diabetes, while ABD+HDL+BP may have a high risk of CVD and MACE when compared to the other MS combinations.

A previous study showed that central obesity increases with age prior to reaching the age of 75 (31). The Longitudinal Aging Study Amsterdam (LASA) found that BMI was an independent predictor of metabolic syndrome among community-dwelling adults aged 55–85 years after a 3-year follow-up (32). However, the study did not exclude participants taking medications for hypertension and diabetes, and only 218 participants were recruited. In our study, we found that the prevalence of metabolic syndrome and central obesity was increased, but BMI was decreased after 2 years of follow-up.

In this study, the four-class LCA model was capable of classifying the subgroups of metabolic syndrome. A past study showed that more components of metabolic syndrome will increase the incidence of new-onset diabetes, but that study did not discuss which combination of metabolic syndrome components is the strongest predictor of new-onset diabetes (13). In a study of older adult Asians who were followed for 5 years, it was found that when taking the older adults without any metabolic syndrome components as the reference group, the HRs of diabetes in three components of metabolic syndrome was 1.79, in four components 2.18, and in five components 3.05 (33). Past studies have only discussed how the more metabolic syndrome components there are, the better the prediction of incident CVD and diabetes (4, 6, 14, 34) there will be. It has also been shown that the risk of developing diabetes differs between metabolic syndrome participants with or without impaired fasting glucose. However, the abovementioned studies did not exclusively investigate older adults and did not exclude patients with hypertension (14, 35). In our study, we found that ABD+GLU+HDL+BP had the highest probability of developing new-onset diabetes and that ABD+HDL+BP was associated with the risk of developing CVD and MACE in a 2-year follow-up. In addition to adjusting a family's history of diabetes, CVD, and healthy behaviors, ABD+HDL+BP develops into CVD and MACE significantly disappears statistically (Supplemental Table S2). It may be said that healthy behavior is an interfering factor for the risk of CVD and that a 2-year follow-up period for the effect of blood sugar on CVD is not enough. To our knowledge, this is the first study to report these findings.

There were several limitations in this study. First, older adults with severe illness or disability cannot participate in the New Taipei Seniors Health Examination program. Second, the physical function status of the older adults participating in the program was not clear, and their status will affect the presentation of metabolic syndrome. Third, similar to previous community-based studies, there was recall bias in marital status, socioeconomic status, education level, personal and family history, medical history, lifestyle factors, and medication use. Fourth, the follow-up period was only 2 years, so events, such as new-onset CVD or stroke, possibly could not be observed based on our subtypes.

## Conclusions

This epidemiological analysis shows that the prevalence of metabolic syndrome and central obesity increases with age. The four-class LCA model was suitable for classifying the metabolic syndrome participants. The metabolic syndrome subtype comprising central obesity + high BP + low HDL + high blood glucose displayed the highest probability of developing new-onset diabetes during the 2-year follow-up of this study. Further longitudinal data and interventional studies are still needed in order to clarify the clinical significance of metabolic syndrome during old age.

## Data Availability Statement

The data that support the findings of this study are available from New Taipei City Government, but restrictions apply to the availability of these data, which were used under license for the current study, and so are not publicly available. Data are however available from the authors upon reasonable request and with permission of New Taipei City Government.

## Author Contributions

C-SL wrote the main manuscript text. H-PL, R-CC, and C-HL performed the data collection. L-KC, W-JL, and S-YL designed the study and edited the critical reviews for whole manuscript. All authors contributed to the article and approved the submitted version.

## Conflict of Interest

The authors declare that the research was conducted in the absence of any commercial or financial relationships that could be construed as a potential conflict of interest.

## Publisher's Note

All claims expressed in this article are solely those of the authors and do not necessarily represent those of their affiliated organizations, or those of the publisher, the editors and the reviewers. Any product that may be evaluated in this article, or claim that may be made by its manufacturer, is not guaranteed or endorsed by the publisher.
