# Supplementary material for: Subtypes of Premorbid Metabolic Syndrome and Associated Clinical Outcomes in Older Adults
Source: Front Med (Lausanne). 2022 Feb 11;8:698728. doi: 10.3389/fmed.2021.698728 (PMC8873979; doi:10.3389/fmed.2021.698728)
Supplement: Supplementary file 1 [file Data_Sheet_1.docx]

| Supplemental Table 1. Baseline characteristics of included and excluded participants in 2014. | | | | | | |
| --- | --- | --- | --- | --- | --- | --- |
|  | 2014 include  (n=36,410) | |  | 2014 exclude (n=4,337) | | *p* value |
|  | n | % |  | n | % |  |
| **Age** | 73.39 | ±6.77 |  | 72.47 | ±7.52 | **<0.001**** |
| **Gender** |  |  |  |  |  | 0.179 |
| Male | 17,464 | (48.0%) |  | 1,914 | (44.1%) |  |
| Female | 19,946 | (54.8%) |  | 2,423 | (55.9%) |  |
| **Smoking** | 2,557 | (7.3%) |  | 269 | (6.4%) | 0.050 |
| **Drinking** | 4,447 | (12.7%) |  | 506 | (12.2%) | 0.319 |
| **Exercise** | 24,942 | (73.0%) |  | 2,979 | (73.8%) | 0.338 |
| **BMI** | 25.09 | ±26.60 |  | 24.46 | ±3.40 | 0.432 |
| Chi-square test or Independent t test. **p*<0.05, ***p*<0.01 | | | | | | |

| Supplemental Table 2. Poor health outcomes among different metabolic syndrome states after adjusted for gender, age, family history of DM, cardiovascular  disease, and health behaviors. | | | | | | | | | | | | |
| --- | --- | --- | --- | --- | --- | --- | --- | --- | --- | --- | --- | --- |
| Metabolic syndrome  state | Poor health outcome | | | | | | | | | | | |
|  | DM | | | Stroke | | | CVD | | | MACE | | |
|  | Number/event | HR | *p* | Number/event | HR | *p* | Number/event | HR | *p* | Number/event | HR | *p* |
| MS component 0 | 570/16 | reference |  | 570/6 | reference |  | 570/62 | reference |  | 570/67 | reference |  |
| MS component 1 | 1,126/53 | 1.29 | 0.376 | 1,126/13 | 1.29 | 0.632 | 1,126/118 | 0.93 | 0.653 | 1,126/130 | 0.95 | 0.738 |
| MS component 2 | 1,355/156 | 3.02 | **<0.001**** | 1,355/21 | 1.48 | 0.439 | 1,355/155 | 0.90 | 0.531 | 1,355/167 | 0.90 | 0.488 |
| MS | 1,486/307 | 5.50 | **<0.001**** | 1,486/24 | 1.51 | 0.409 | 1,486/191 | 0.97 | 0.870 | 1,486/211 | 1.01 | 0.939 |
| ABD HDL BP | 79/4 | 1.95 | 0.251 | 79/1 | 1.46 | 0.739 | 79/14 | 1.76 | 0.079 | 79/15 | 1.73 | 0.076 |
| ABD GLU HDL BP | 149/46 | 7.15 | **<0.001**** | 149/2 | 1.39 | 0.698 | 149/21 | 0.74 | 0.310 | 149/23 | 0.79 | 0.388 |
| ABD GLU HDL BP TRI | 126/34 | 7.53 | **<0.001**** | 126/1 | -- |  | 126/22 | 1.22 | 0.470 | 126/22 | 1.14 | 0.640 |
| ABD GLU BP | 384/80 | 5.26 | **<0.001**** | 384/6 | 1.23 | 0.756 | 384/66 | 1.07 | 0.720 | 384/69 | 1.05 | 0.806 |
| Others MS | 748/143 | 5.24 | **<0.001**** | 748/14 | 1.80 | 0.274 | 748/68 | 0.92 | 0.673 | 748/82 | 1.01 | 0.975 |
| Adjusted for gender, age, family history of DM and cardiovascular disease, health behaviors. | | | | | | | | | | | | |
